# Non-walled spherical *Acinetobacter baumannii* is an important type of persister upon *β*-lactam antibiotic treatment

**DOI:** 10.1080/22221751.2020.1770630

**Published:** 2020-06-02

**Authors:** Jin Zou, Si-Hoi Kou, Ruiqiang Xie, Michael S. VanNieuwenhze, Jiuxin Qu, Bo Peng, Jun Zheng

**Affiliations:** a Faculty of Health Sciences, University of Macau, Macau SAR, People’s Republic of China; b Department of Molecular and Cellular Biochemistry, Indiana University, Bloomington, IN, USA; c Department of Clinical Laboratory, The Third People’s Hospital of Shenzhen, Southern University of Science and Technology, National Clinical Research Center for Infectious Diseases, Shenzhen, People’s Republic of China; d School of Life Sciences, Sun Yat-sen University, Guangzhou, People’s Republic of China; e Laboratory for Marine Biology and Biotechnology, Qingdao National Laboratory for Marine Science and Technology, Qingdao, People’s Republic of China; f Institute of Translational Medicine, University of Macau, Macau SAR, People’s Republic of China

**Keywords:** *Acinetobacter baumannii*, persisters, drug tolerance, non-walled spherical bacterium, *β*-lactam antibiotics

## Abstract

Bacterial persistence is one of the major causes of antibiotic treatment failure and the step stone for antibiotic resistance. However, the mechanism by which persisters arise has not been well understood. Maintaining a dormant state to prevent antibiotics from taking effect is believed to be the fundamental mechanistic basis, and persisters normally maintain an intact cellular structure. Here we examined the morphologies of persisters in *Acinetobacter baumannii* survived from the treatment by three major classes of antibiotics (i.e. *β*-lactam, aminoglycoside, and fluoroquinolone) with microcopy and found that a fraction of enlarged spherical bacteria constitutes a major sub-population of bacterial survivors from *β*-lactam antibiotic treatment, whereas survivors from the treatment of aminoglycoside and fluoroquinolone were less changed morphologically. Further studies showed that these spherical bacteria had completely lost their cell wall structures but could survive without any osmoprotective reagent. The spherical bacteria were not the viable-but-non-culturable cells and they could revive upon the removal of *β*-lactam antibiotics. Importantly, these non-walled spherical bacteria also persisted during antibiotic therapy *in vivo* using *Galleria mellonella* as the infection model. Additionally, the combinational treatment on *A. baumannii* by *β*-lactam and membrane-targeting antibiotic significantly enhanced the killing efficacy. Our results indicate that in addition to the dormant, structure intact persisters, the non-wall spherical bacterium is another important type of persister in *A. baumannii.* The finding suggests that targeting the bacterial cell membrane during *β*-lactam chemotherapy could enhance therapeutic efficacy on *A. baumannii* infection, which might also help to reduce the resistance development of *A. baumannii*.

## Introduction

The treatment failure on bacterial infection diseases has become a global crisis again only less than 100 years after the first antibiotic was introduced for clinical use. On the one hand, the mis- or over-use of antibiotics in the past several decades have led to the rapid evolution of antibiotic resistance in almost all the major bacterial pathogens, resulting in an increasing scarcity of an effective antibiotic. On the other hand, bacterial persistence has augmented the difficulties in the effective treatment by the limited source of antibiotics. Different from antibiotic resistance, bacterial persisters are a sub-population of isogenic bacteria that can survive the prolonged exposure to high concentration of antibiotics without generating any genetic variation to counteract the bactericidal effects of the antibiotics. Bacterial persisters were first identified in 1944 by Joseph Bigger, who found that a small portion of *Staphylococcus* population was not killed by penicillin but still kept the susceptibility to the same antibiotic after re-growth [1]. Although bacterial persistence has been noticed for seven decades, it was appreciated only recently as one of the major causes of antibiotic recalcitrance and relapse of infection diseases [2–5].

In addition to resulting in the chronic bacterial infections, persisters can provide a viable cell reservoir from which resistant mutant can emerge by *de novo* chromosomal mutation or horizontal gene transfer, functioning as a stepping stone for the evolution of antibiotic resistance [6–8]. Furthermore, persistence is also able to promote the evolution of antibiotic resistance by increasing the mutation rates [9]. Therefore, an effective eradication of bacterial persisters will not only prevent treatment failure of chronic infection but also alleviate the development of antibiotic resistance. Understanding the underlying mechanism of persistence formation will aid the development of such effective therapeutic approach.

While the clinical implications of bacterial persistence have been well demonstrated [10], the mechanisms by which persisters arise and the basis for drug tolerance are yet to be fully elucidated [11,12]. Cell dormancy has long been considered as the fundamental mechanism for the formation of persisters. In this model, antibiotics cannot exert their lethal effects even after they bind to their targets, because the bacteria are in a dormant state and the downstream pathways of antibiotic-drug interaction are inactive. Such dormant persisters maintain an intact cellular structure [5]. Several factors, such as toxin-antitoxin module, (p)ppGpp, SOS response pathways and intracellular ATP level, contribute to the dormancy of the cells [4]. Additionally, actively pumping out the intracellular antibiotics by efflux pump also facilitate the dormancy of bacterial persisters [13,14].

*Acinetobacter baumannii* is a gram-negative coccobacillus that is aerobic, pleomorphic and non-motile. It has emerged as a highly troublesome pathogen responsible for a broad range of nosocomial infections, including ventilator-associated pneumonia and bloodstream infections with a high mortality rate [15]. In addition, *A. baumannii* is also a causative agent for skin and soft tissue infections, secondary meningitis and wound infections [16]. Importantly, *A. baumannii* has remarkable ability to acquire resistance. In fact, it has developed resistance to almost all known antibiotics, and the multiple drug resistance (MDR) has also been widely documented [17,18]. The clinical importance of *A. baumannii* was emphasized recently by World Health Organization (WHO), and *A. baumannii* was selected as the priority 1 (critical) pathogen among its “priority pathogens” list for research and development related to new antibiotics [19]. In addition to the widespread of drug resistance, *A. baumannii* persistence also contributes to the treatment failure even at high doses of antibiotics [20]. Despite the endeavours have been made to understand the persistence and its eradication [21–24], the mechanism for persistence formation in *A. baumannii* is yet to be fully understood.

In this study, we investigated the morphologies of *A. baumannii* persisters produced by different antibiotics and found a major faction of enlarged spherical cells presenting in persisters survived from *β*-lactam antibiotic killing. Subsequently, we found that *β*-lactam antibiotics efficiently inhibited the cell wall synthesis in *A. baumannii* and formed non-walled spherical cells. The non-walled spherical *A. baumannii* demonstrated enlarged cellular size and was able to persist under both *in vitro* and *in vivo* conditions as a new type of persisters. Treatment of *A. baumannii* by the combination of *β*-lactam and cell membrane-targeting reagent enhanced the killing of the pathogens. Our study identifies a new type of persisters featured by active metabolism and damaged cellular structure and provides a promising approach for the efficient treatment of *A. baumannii* infection.

## Materials and methods

### Bacterial strains, plasmids and growth conditions

The bacterial strains and plasmids used in this study are listed in Table S1. LB broth was used for all the experiments except for combinational killing assay, in which cation-adjusted Mueller–Hinton broth (MHB) medium was used. Culture media were supplemented with 10 μg/mL tetracycline when necessary.

### Bacterial strain construction

The strain containing *dnak::*msfGFP translational fusion was constructed by *Sac*B-mediated allelic exchange following previous description [25] with the suicide plasmid pEXG2 harbouring a fragment of *dnak* gene, *msfGFP* gene which was amplified from the template plasmid of pWM91_VipA-msfGFP [26] and a fragment of DNA sequence downstream of *dnaK* gene on the chromosome. Primers used are listed in Table S2.

### Isolation of persisters and bacterial killing assay

The bacterial killing was performed as described previously [27] and a detailed protocol is provided in supplementary experimental procedures.

### Fluorescent microscopy

Bright field and fluorescence imaging were performed on an inverted microscope (Carl Zeiss Axio Observer Z1). Appropriate filter sets were selected for each dye according to their excitation and emission spectra. HADA was imaged in DAPI channel and false-coloured in red. Flow Cell System FCS2 (The Focht Chamber System 2, Bioptechs) was used to record prolonged bacterial killing and re-growth. Detailed information is described in supplementary experimental procedures.

### Image acquisition and analysis

All microscopy images were analysed by ImageJ software (Fiji). The bacterial surface area was measured from bright-field images and the pixel intensity within a cell was measured from the fluorescence emission channel.

### *Galleria mellonella* infection model

*G. mellonella* caterpillars in the final-instar larval stage were used in this study and infections were conducted, as described previously [28,29]. Detailed information is described in supplementary experimental procedures.

### Determination of the fractional inhibitory concentration index (FICI)

The FICI was determined, as described previously [30]. Detailed information is provided in supplementary experimental procedures.

## Results

### *A. baumannii* persisters from antibiotic killing demonstrates distinct morphotypes

**Figure 1.**
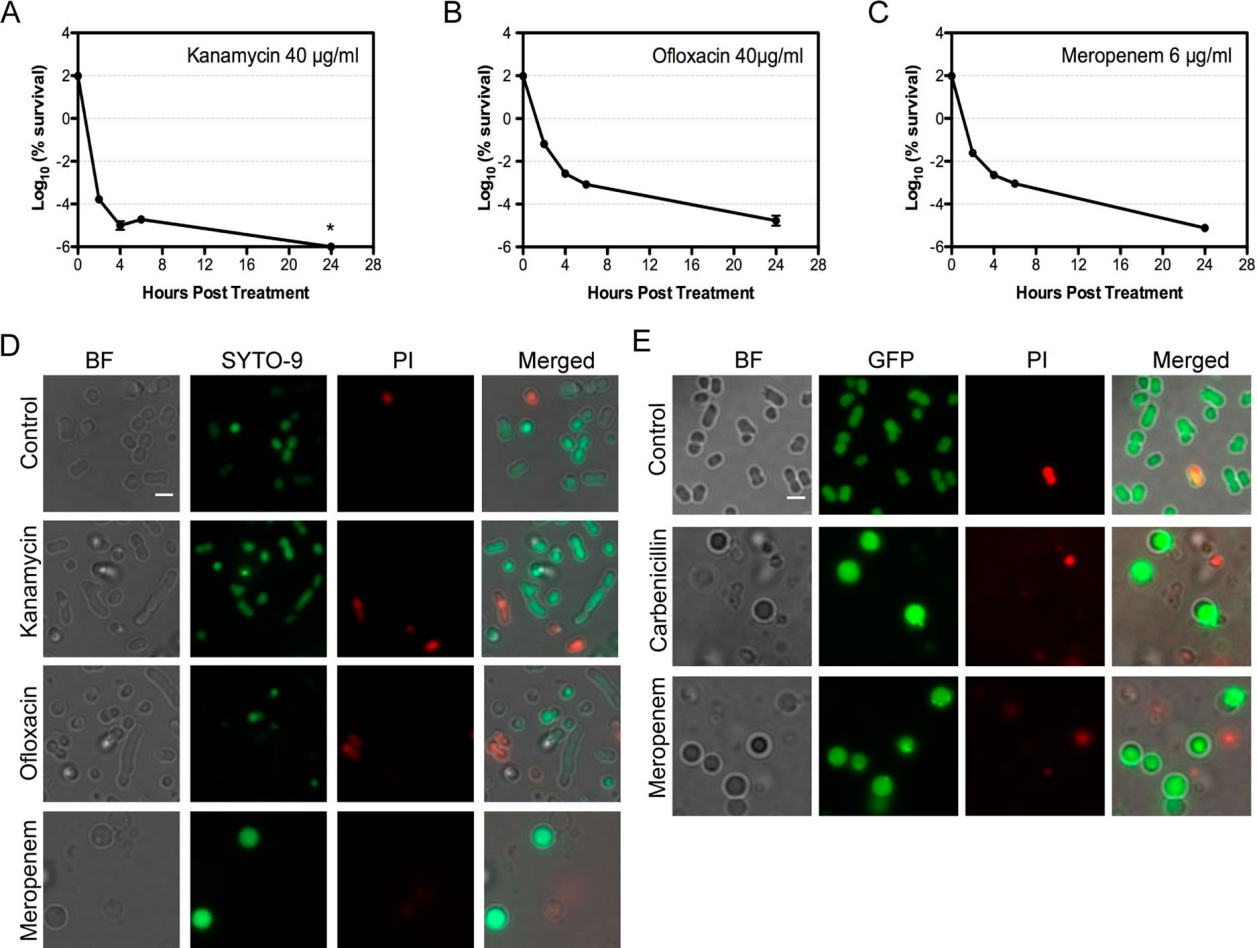
*A. baumannii* persisters from various antibiotics show distinct morphotypes. (A–C) Biphasic killing curves of *A. baumannii* ATCC17978 under the treatment of 40 μg/mL kanamycin (A), 40 μg/mL ofloxacin (B) and 6 μg/mL meropenem (C). (D) The fluorescent microscopy showing distinct morphotypes of *A. baumannii* survivors. Bacteria were treated with 40 μg/mL kanamycin, 40 μg/mL ofloxacin or 6 μg/mL meropenem at 37°C for 4 h and then stained with SYTO-9 and propidium iodide (PI). (E) The survivors of *A. baumannii dnak*::*msfGFP* show a similar spherical shape after treatment by various *β*-lactam antibiotics. Bacteria were treated with 150 μg/mL carbenicillin or 6 μg/mL meropenem at 37°C for 4 h and then stained with PI for microscopy. (Scale bar, 2 μm). *: under detect limitation. BF: Bright field.

We attempted to investigate the persistence in *A. baumannii* by conducting a dose-dependent antibiotic killing on *A. baumannii* ATCC17978 with a representative from the three major classes of antibiotics, namely meropenem (*β*-lactam), kanamycin (aminoglycoside) and ofloxacin (fluoroquinolone) (Fig. S1A–S1C). A concentration of antibiotic that kills about 99.99% of bacteria after 4 h incubation was chosen for each of these antibiotics: 6 µg/mL for meropenem, 40 µg/mL for kanamycin and 40 µg/mL for ofloxacin. Bacterial cultures treated with such a concentration of individual antibiotic demonstrated a classic biphasic killing curve, a characteristic for bacterial persisters (Figure 1(A–C)) [31,32]. The frequency of persisters obtained from the bacterial culture was 5.3 × 10^−7^ for kanamycin, 8.5 × 10^−5^ for ofloxacin and 2.2 × 10^−5^ for meropenem.

The untreated *A. baumannii* demonstrated pleomorphism: the majority cells showed a short rod morphotype, and a small portion of bacteria resembled the coccobacilli or the long rod bacteria (Figure 1(D)). Upon treatment by antibiotics, most of bacteria were killed, as few bacteria could be visualized under microscope in the bacterial culture without being concentrated. The morphotype of the observed bacteria treated by kanamycin or ofloxacin was less changed (Figure 1(D)), though an increasing number of longer rod cell were observed in bacteria treated with ofloxacin. In contrast, a significant number of bacteria treated by lethal concentration of meropenem demonstrated a dramatic morphological change: the cells turned into spherical shape and got enlarged (Figure 1(D)). This is in consistent with the previous observation [33]. We stained the bacteria with SYTO-9 and propidium iodide (PI). Our results showed that the majority of cells observed under microscope were both SYTO-9 positive and PI negative, including the spherical *A. baumannii* produced by meropenem treatment, suggesting that the cells observed are still viable.

For direct visualization the cell morphotypes *in vitro* and *in vivo*, we engineered a chromosomal *msfGFP* fusion at the C-terminal of DnaK in *A. baumannii* ATCC17978 to produce the strain of *dnak*::*msfGFP*. DnaK was known to be expressed during antibiotic treatment in *A. baumannii* [34]. Bacteria with DnaK::msfGFP can be easily visualized under microscope. The bacterial derivative demonstrated a similar response to meropenem (Fig. S1D). Similarly, the treatment of *A. baumannii* with 150 µg/mL carbenicillin resulted in a killing curve that bacterial survivors at 4 h post-treatment could largely represent the persister cells (Fig. S1E and S1F). Bacterial survivors from carbenicillin-treated *dnak*::*msfGFP* culture showed a similar spherical shape as those treated by meropenem (Figure 1(E)) and were distinct from the bacteria untreated or those treated by kanamycin or ofloxacin (Figure 1(D)). Extension on the incubation time with the carbenicillin or meropenem up to 11 h or 24 h did not eliminate them from the culture (Fig. S1G and S1H). These results suggest that the spherical cells are a common morphotype for the bacteria that survive the killing by lethal concentration of *β*-lactam antibiotics.

### Spherical *A. baumannii* is persisters that can revive

**Figure 2.**
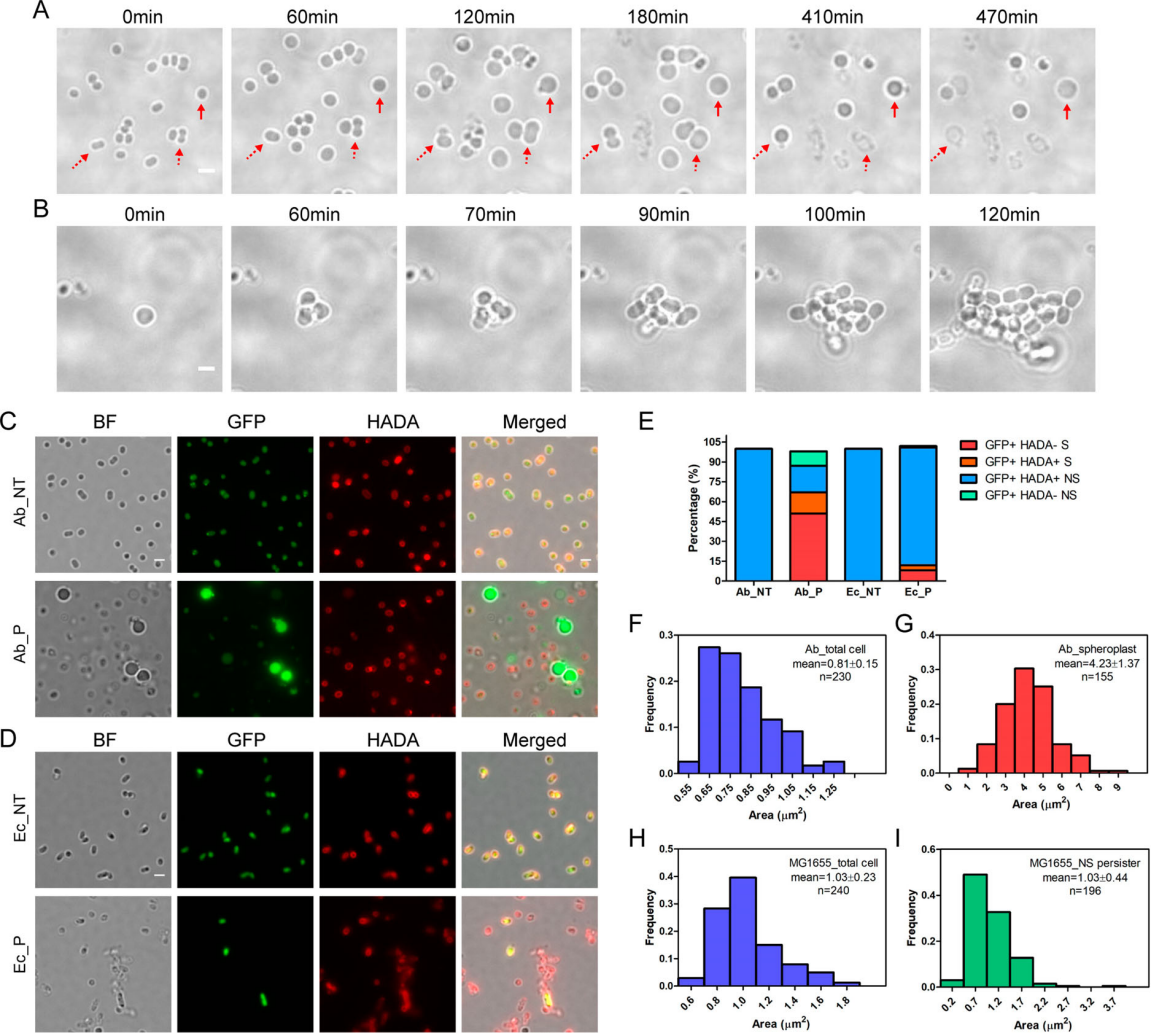
Characteristic of spherical persisters of *A. baumannii* induced by meropenem. (A) Time-lapse microscopy showing that spherical cells survived from 10 μg/mL meropenem in LB media (from movie S1). Dashed arrows showing the cell escaped from one point and finally got burst. Solid arrow pointing to the cell that increased in cell size and still persisted at the end of observation. (B) Time-lapse microscopy showing that isolated spherical persister cells regrew in LB media upon the antibiotic removal (from movie S2). (C) Fluorescent microscopy on overnight culture of *dnaK::msfGFP* stained with HADA without (upper, Ab_NT) or with the treatment of 6 μg/mL of meropenem for 4 h (lower, Ab_P). (D) Fluorescent microscopy of *E. coli* MG1655 expressing eGFP stained by HADA without (upper, Ec_NT) or with the treatment of 6 μg/mL of meropenem for 4 h (lower, Ec_P). (E) Percentage of various cell types in the total cells of *dnaK::msfGFP* observed in (C) or of *E. coli* MG1655 expressing eGFP observed in (D). GFP+: GFP signal positive; HADA+/−: HADA signal positive or negative; S: spherical cell; NS: non-spherical cells. (F–G) Histogram of cell surface area of *dnaK::msfGFP* before (F) and after treatment (G) in the experiment of (C). (H–I) Histogram of cell surface area of *E. coli* MG1655 expressing eGFP before (H) and after treatment (I) in the experiment of (D). (Scale bar, 2 μm). BF: Bright field.

To confirm that spherical bacteria are bona fide persisters that can revive upon the removal of antibiotics, rather than the viable-but-non-culturable cells (VBNCs), we monitored the formation of spherical bacteria upon meropenem treatment and their re-growths after the removal of antibiotics with FCS2 device, a temperature-controlled flow cell system. We found that bacteria become spherical after 60 min antibiotic treatment and simultaneously the size of bacterial cells started to increase. Time-lapse microscopy showed that spherical cells first protruded from one side or two sides of the bacteria, likely the broken site of the cell wall, and then the whole cell escaped from one point and eventually formed the spherical cell. As the size increased, some of the spherical cells got burst. However, a significant number of the enlarged spherical cells still persist after 470 min post-antibiotic treatment (Figure 2(A) and Movie S1). We also noticed that a transient protrusion occurred from some of the spherical enlarged cells and then the cell size got reduced or burst.

The ability of the spherical bacteria to divide upon the removal of *β*-lactam antibiotics was then examined. *A. baumannii* culture was firstly treated with 10 µg/mL meropenem for 4 h, followed by washing to remove the residual antibiotics. Bacterial survivors were then concentrated and observed by time-lapse microscopy. The fresh LB medium was provided to these cells using the FCS2 device during observation. Consistently, meropenem treatment produced the spherical bacteria with enlarged size. Time-lapse imaging of the spherical bacteria showed that they started to divide at 60 min after the fresh LB medium was provided and eventually formed colonies (Figure 2(B) and Movie S2). These results suggested that the spherical bacteria produced by *β*-lactam antibiotics are viable and re-growable, but not VBNCs. The re-grown bacteria were still sensitive to the same antibiotic and turned into spherical morphotype from the normal cells upon treatment (Fig. S2A and S2B). Importantly, the isolated persisters demonstrated a comparable minimum inhibition concentration (MIC_50_) for each antibiotic with that of bacteria before antibiotic treatment (Fig S3 and Table S3). Our results collectively suggest that the isolated persisters did not acquire any resistance. Thus, the spherical *A. baumannii* is a new type of persister.

### Spherical persisters completely lose their cell walls

We next asked whether the spherical bacteria observed were those lost the cell wall or simply dormant cells that changed their morphotypes. We employed the fluorescent D-amino acid analogue HCC-amino-D-alanine (HADA) to label the peptidoglycan (PG) of the cell walls in *A. baumannii dnak*::*msfGFP* that expressed GFP. HADA can be specifically incorporated into PG and can be widely used for probing the presence of bacterial cell wall in other bacterial species [33,35]. Similarly, HADA was incorporated into both exponential and stationary phases of *A. baumannii* (Fig. S4). We found that nearly 100% of the *A. baumannii dnak*::*msfGFP* or *Escherichia coli* (expressing eGFP) grown in LB media are labelled by HADA (Figure 2(C,D), upper panel). The bacteria gave both green and red fluorescence, indicating that they are live bacteria with an intact cellular structure. Upon meropenem exposure, most *A. baumannii* turned dim or became negative in green fluorescence, while were still positive in red fluorescence, suggesting that the bacteria are dead and cytoplasm might have been released, left an empty cell wall skeleton. However, we observed that the spherical persisters were positive in green fluorescence but negative in red fluorescence, suggesting that these persisters lost their cell wall completely (Figure 2(C), lower panel). Similarly, almost all *E. coli* treated by meropenem became green fluorescence negative, suggesting that the cells are dead. The few remaining *E. coli* positive in both green and red fluorescence resembled the same rod shape morphotype as those untreated bacteria (Figure 2(D), lower panel), suggesting that the persisters in *E. coli* are mainly dormant persisters with an intact cellular structure.

The ratio of spherical bacteria was then quantified under microscope. About 68.6% of persistent cells in *A. baumannii* culture after treatment by meropenem were spherical with an enlarged bacterial size, in contrast to only ∼8.7% of spherical bacteria in *E. coli*. Furthermore, 51.8% of the spherical persisters of *A. baumannii*, compared to 8.2% of *E. coli*, were totally negative in HADA labelling. In addition to the spherical persisters, ∼31.4% of persisters in *A. baumannii* exhibited the same non-spherical rod shape as untreated bacteria, and 20.4% of them were HADA positive, suggesting that they are likely the dormant persisters. In contrast, ∼91.2% *E. coli* persisters in cultures treated by meropenem remained the same rod shape as untreated, among which 89.5% of them were HADA positive (Figure 2(C–E)).

To quantify the increases in bacteria size observed in spherical cells, the surface area of *A. baumannii* perisisters from meropenem treatment was analysed by ImageJ (Fiji). *A. baumannii* grown in LB media demonstrated an average surface area of 0.81 ± 0.15 μm^2^ (Figure 2(F)). In contrast, the surface area of non-walled spherical persisters in *A. baumannii* treated with meropenem increased dramatically, reaching an average of 4.23 ± 1.37 μm^2^ (Figure 2(G)). However, the persister of *E. coli*, mainly in non-spherical rod shape, and produced after the treatment with meropenem, showed a similar surface area (1.03 ± 0.44 μm^2^) as untreated (1.03 ± 0.23 μm^2^) (Figure 2(H–I)).

### *A. baumannii* forms spherical persisters *in vivo*

**Figure 3.**
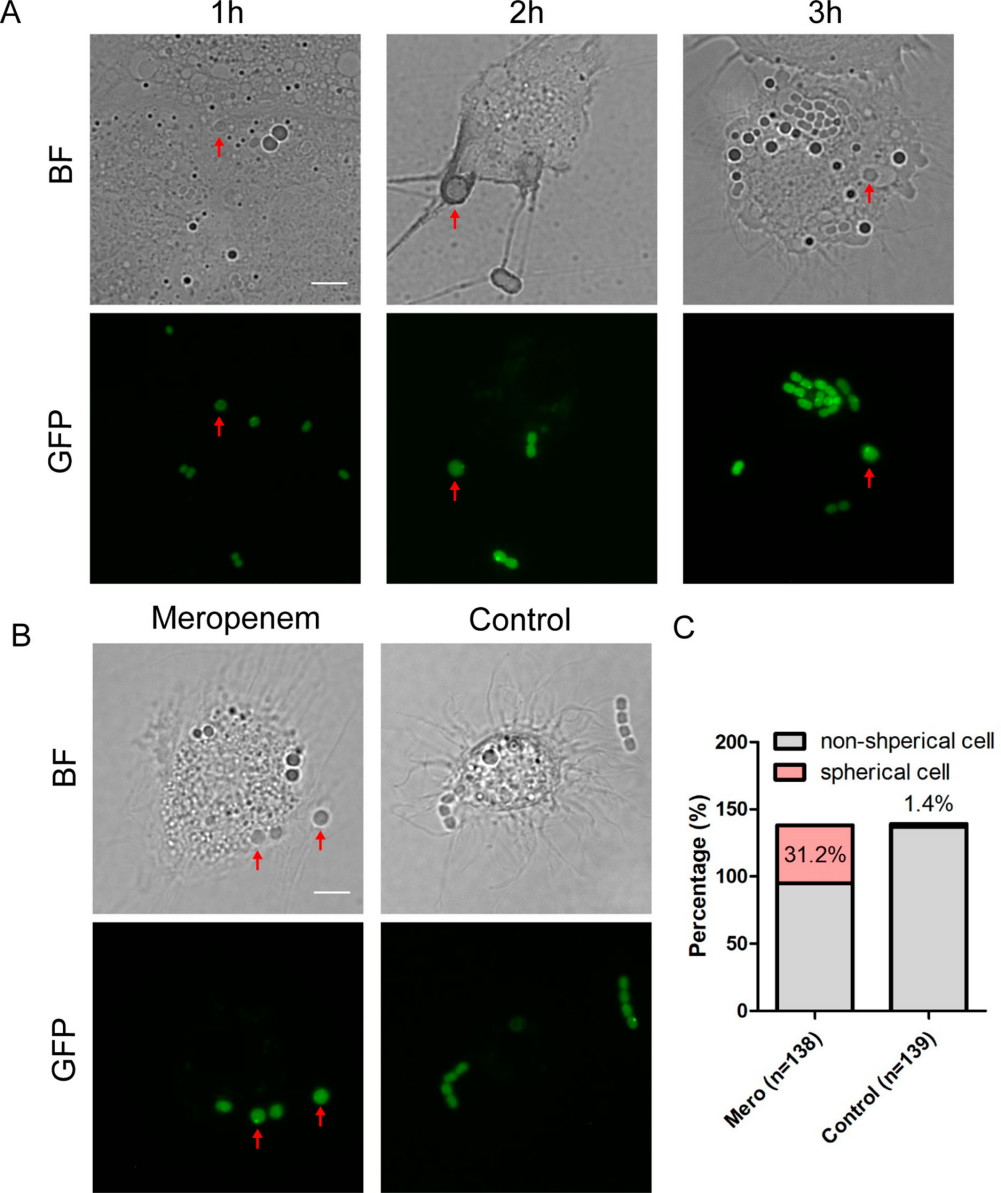
*A. baumannii* forms spherical persisters in the host environment. (A) The morphotypes of *A. baumannii* in the *G. mellonella* larvae without antibiotic treatment. Bright-field and the corresponding fluorescent images of haemocytes extracted at 1 h, 2 h and 3 h after the larvae being infected by *dnaK::msfGFP*. The red solid arrows pointing the spherical cells. (B) The morphotypes of *A. baumannii* in the *G. mellonella* larvae upon treatment with meropenem. The larvae were infected by *dnaK::msfGFP* for 1 h, followed by treatment with 6 mg/kg of meropenem (left) or filtered water (control, right) for another 30 min. Haemocytes were then extracted for bright-field and fluorescent imaging. (Scale bar, 5 μm). The red solid arrows pointing the spherical cells. (C) Percentage of spherical and non-spherical bacterial cells in the extracted haemocytes from the infected larvae treated with meropenem or control in (B). BF: Bright field.

We next asked whether the non-walled enlarged spherical *A. baumannii* was produced and persisted in host environment. We infected larvae of *G. mellonella*, a widely used animal model for studying the pathogenesis and antimicrobial treatment involved in *A. baumannii* [28,36,37], with *dnak*::*msfGFP*. After 3 h infection with *A. baumannii*, the haemolymph of *G. mellonella* was extracted every hours from larvae for observation with fluorescent microscope. Most of *A. baumannii* cells in haemolymph isolated from untreated *G. mellonella* were in short rod shape with normal size, suggesting that the bacteria maintain an intact cell wall. A few *A. baumannii* cells showed spherical shape with increased cell size (Figure 3(A)). These enlarged spherical *A. baumannii* are likely produced by lysozyme in the haemolymph, which has been shown to produce non-walled L-form in *Bacillus subtilis* [29]. To test if *β*-lactam antibiotics could enhance the ratio of spherical cells *in vivo*, we treated the larvae with meropenem at 1 h after infection by *dnak*::*msfGFP*. At 30 min post treatment, the haemolymph was extracted, and the bacteria were examined under microscope. Compared to bacteria in untreated haemolymph, an increased number of bacteria isolated from the treated *G. mellonella* showed a spherical shape: 31.2% of bacterial survivors are spherical shape compared to 1.4% in the controlled group (Figure 3(B,C)). Therefore, spherical *A. baumannii* persisters could be formed during the therapeutic process in the host environment upon treated by *β*-lactam antibiotics.

### Membrane targeting agents enhance the killing of *β*-lactam antibiotics

**Figure 4.**
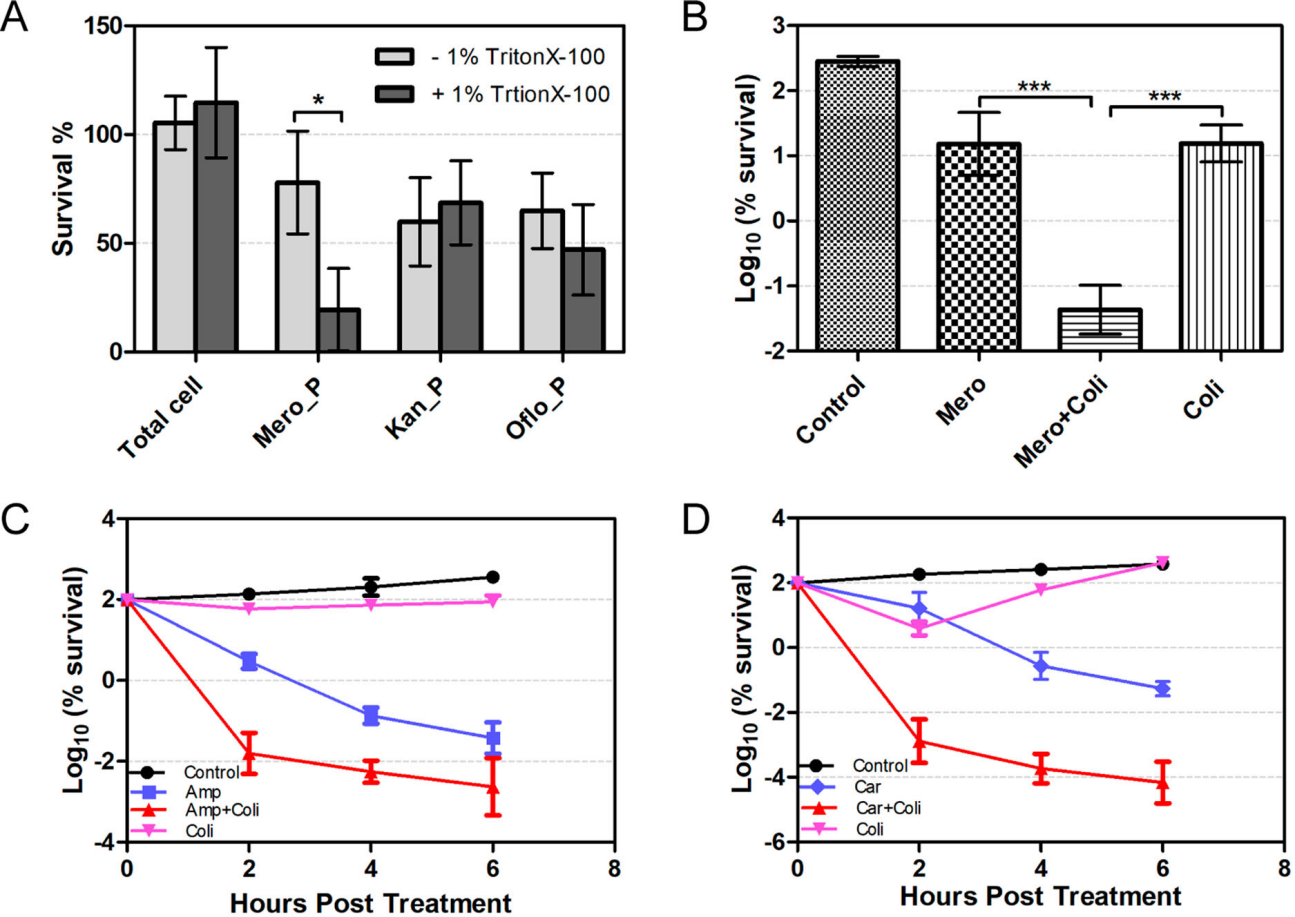
Synergistic effect of *β*-lactam antibiotics and membrane damage agents on the killing of *A. baumannii.* (A) The effect of 1% Trition X-100 on the survival of bacterial persisters from the treatment of meropenem (6 μg/mL) or kanamycin (40 μg/mL) or ofloxacin (40 μg/mL). (B–D) The combination of *β*-lactam antibiotics and colistin enhances the killing on *A. baumannii*. Bacteria were treated by the combination of 0.5 μg/mL of meropenem and 2 μg/mL of colistin for 1h (B) or for a time-course treatment by 30 μg/mL of carbenicillin and 2 μg/mL of colistin (C) or 200 μg/mL of ampicillin and 2 μg/mL of colistin (D). The values represent the mean ± SD from of three independent experiments (*: *p* value <0.05 ***: *p* value <0.001).

Since the spherical persisters have lost their cell walls, we hypothesized that the addition of membrane-damage agent during *β*-lactam antibiotic treatment could produce a synergistic effect and enhance bacterial killing by *β*-lactam antibiotics. A low concentration (1%) of Triton X-100, a common non-ionic detergent that damages cell membrane, alone does not affect the survival of untreated *A. baumannii*. However, its addition to *A. baumannii* meropenem treatment enhances the killing potency of the antibiotic by about 50%. In contrast, addition of 1% Triton X-100 *A. baumannii* during the treatment by kanamycin or ofloxacin did not produce any synergistic effect (Figure 4(A)).

Colistin is the last-resort antibiotic for multidrug-resistant gram-negative organisms including *A. baumannii*, and it targets the lipopolysaccharide and can dissolve bacterial membrane. However, it has nephrotoxicity and neurotoxicity at high dose [38,39]. We found that a low concentration of colistin (2µg/mL), which had little killing effect on bacteria by itself, could sufficiently enhance the killing potency of meropenem by over 100-fold (Figure 4(B)). Importantly, the synergistic effect was observed not only between colistin and meropenem, but also with other *β*-lactam antibiotics (i.e. ampicillin and carbenicillin), with an increasing in killing on *A. baumannii* by a range of 100- to 1000-fold (Figure 4(C,D)). Furthermore, the enhanced killing efficiency was also observed when the combination of carbenicillin and colistin was used to treat two clinical isolates (Iso 03126 and Iso 03368) of *A. baumannii* (Fig. S5A and S5B). The synergistic effect of the combination of colistin and *β*-lactam antibiotics on bacterial growth was also investigated by fractional inhibitory concentration index (FICI), and the combination of colistin with ampicillin or meropenem showed significant synergistic effect, whereas no significant synergistic effect between colistin and carbenicillin was observed (Table S4). Considering that FICI reflects the inhibitory but not bactericidal effect of the drugs at low concentration, whereas the killing assay by CFU enumeration examines their bactericidal effect at high concentration, it is thus not surprised to see the dramatic potentiation of colistin on *β*-lactam killing but less significant in enhancing the inhibition of bacteria growth in the FICI assay.

## Discussion

Bacterial persistence is one of the major causes of antibiotic treatment failure and infection relapse. Dormancy has been recognized as the basic mechanism for the persistence [2–4]. The inactive metabolism in dormant bacteria results in the failure of antibiotic accumulation to an effective concentration that can cause enough damage to bacterial cells for a lethal effect. In this report, we found that the treatment of *A. baumannii* with *β*-lactam successfully damaged/inhibited the bacterial cell wall and produced the non-walled, enlarged spherical cells. Our results suggest that the non-walled, enlarged spherical cells represent another type of bacterial persisters other than the dormant persisters – they are metabolically active (as demonstrated by the cell wall being damaged) and are able to survive without intact cell wall.

Persisters were less studied until 1998, when Falla and Chopra isolated a mutation in the *hipA*, termed *hipA7*, that enhanced the persistence formation by 100–1000 times [40]. HipA is the toxin of toxin-antitoxin module HipBA [41]. Under stress condition, HipA was activated to halt protein translation and bacterial growth, resulting bacteria into a physiologically dormant state [42–45]. Subsequently, the involvement of several other toxin-antitoxin modules in the persistence formation was shown although there are some instances of counter-evidence challenging their roles [46]. In addition to triggering bacteria to dormancy by activating toxin, lowering intracellular ATP levels and enhancing protein aggregates accumulation both increased the formation of persistence [47,48]. Complementary to driving bacteria to dormancy, enhancing the activity of efflux pump was also demonstrated to facilitate the persistence [14]. Accumulatively, the strategy for persistence used by dormant persisters is to lower its metabolic activities and prevent the antibiotic from effectively damaging their targets so that bacteria can survive from killing and persist. Such persisters maintain an intact cellular structure.

However, our study suggests that different type of persisters likely exist: these types of persisters are metabolically active as their cellular structures have been damaged. Such persisters can be generated by cell wall targeting agent. *β*-lactam is a group of antibiotics that inhibit the catalytic enzymes involved in cell wall synthesis, named penicillin-binding proteins (PBPs), resulting in cell wall-deficient spheroplast. The non-walled spheroplast is liable to lysis driven by high internal osmotic pressure present in bacteria, defining the killing mechanism of *β*-lactam antibiotics. In our study, *β*-lactam antibiotics have successfully inhibited the cell wall synthesis, producing the non-walled spherical bacteria. However, different from *E. coli* a significant number of non-walled spherical *A. baumannii* bacteria were still able to survive both *in vitro* and *in vivo*. Apparently, non-walled spherical *A. baumannii* were under high internal osmotic pressure, judged by the enlarged cellular size visualized under microscope.

Although cell wall is vital for the survival of bacteria, several bacterial species can survive and proliferate without cell wall, named L-form [49]. L-form was mostly achieved artificially by growing them on osmoprotective media in the presence of high levels of antibiotics targeting the enzymes for cell wall synthesis [29,49]. Different from L-form, the non-walled spherical *A. baumannii* produced by *β*-lactam antibiotics survives in the media without any osmoprotective agent and was lack of an obvious bacterial proliferation under our experimental conditions. Consistently, it has been noticed previously that inhibition on the cell wall synthesis in several bacterial species does not always lead to cell lysis when they were grown in media without any osmoprotective agent [33,50–52], suggesting that the non-walled spherical bacteria are a common morphological adaptation to *β*-lactam antibiotic killing. We classify these non-walled spherical *A. baumannii* generated by *β*-lactam antibiotics as persisters because they were a major faction in the bacterial survivors that typically represent the persisters defined based on the classic biphasic killing curve [5]. The survival of these cells was not due to the enhanced tolerance to antibiotics. Tolerance is a feature homogeneously beard by all the individual cells in the bacterial population, whereas persistence is a trait only found in a fraction of cells in such a population [31]. Even though most cells in *A. baumannii* could form non-wall spherical structure, a majority of them quickly burst and only a fraction of them survive. In addition, the non-walled spherical persisters are different from the dormant persisters, as they are metabolically active, and the antibiotics have taken full effect on their targets. In fact, we also observed dormant persisters with intact cellular structure in the fraction of *A. baumannii* persisters produced by meropenem killing, suggesting that the persisters of *A. baumannii* after *β*-lactam antibiotic killing are a population of mixture with heterogenous morphotypes resulting from distinct generating mechanisms.

Cell wall is instrumental for bacteria to prevent from bursting by high internal osmotic pressure. Previous studies showed that the outer membrane of stress-bearing bacteria contributes to the intact of non-walled bacterial cells [53,54]. It is unknown yet what makes the cell membrane of the non-wall spherical *A. baumannii* more elastic or stiffer to sustain high internal osmotic pressure so that cells can survive. Upregulation of fatty acid biosynthesis was known to be critical for the proliferation of L-form [55]. Consistently, inhibition on fatty acid synthesis by cerulenin apparently abolished the formation of spherical bacteria in *A. baumannii* (Fig. S6A), and the expression of A1S_0080 and A1S_0522, encoding putative *β*-Ketoacyl-ACP synthase I and II (FabB/F), respectively, was highly enhanced during meropenem treatment (Fig. S6B). Therefore, excess membrane synthesis, as observed for L-form, is likely one of the strategies for the non-wall spherical *A. baumannii* to survive, which needs further investigation. Since membrane is vital for bacteria from lysis after they lose the cell wall, interruption on the cell membrane would produce synergistic effect on the killing of bacterial pathogens. Colistin, a membrane targeting antibiotic being used for clinical chemotherapy, demonstrated a potent activity to enhance the bacterial killing of *β*-lactam antibiotics *in vitro*. The eradication of *A. baumannii* persisters by the combination of various antibiotics has been well studied recently [56]. While the combination of colistin and aminoglycoside amikacin demonstrated superior clearance on *A. baumannii* persisters, the combination of colistin with *β*-lactam imipenem also significantly reduced the persisters [56], which is consistent with our results. Thus, colistin might be a good candidate for combinational therapy with *β*-lactam antibiotics to treat *A. baumannii* infection. This result is also consistent with our previous studies that targeting bacterial persistence or tolerance could highly enhance the antibiotic treatment [27,57].

In summary, we have identified the non-walled, enlarged spherical *A. baumannii* as a major fraction of bacterial persisters. These persisters are featured by active metabolism and damaged cellular structure, and our results suggest that the combination of *β*-lactam with membrane targeting antibiotic(s) might be a good strategy for the treatment of *A. baumannii* infection.

## Supplementary Material

Supplemental Material

## References

[1] Bigger JW. Treatment of Staphylococcal infections with penicillin by intermittent sterilisation. The Lancet. 1944;244(6320):479–500. doi: 10.1016/S0140-6736(00)74210-3

[2] Hall-Stoodley L, Costerton JW, Stoodley P. Bacterial biofilms: from the natural environment to infectious diseases. Nat Rev Microbiol. 2004 Feb;2(2):95–108. doi: 10.1038/nrmicro821 15040259

[3] Lewis K. Persister cells. Annu Rev Microbiol. 2010;64:357–372. doi: 10.1146/annurev.micro.112408.134306 20528688

[4] Gollan B, Grabe G, Michaux C, et al. Bacterial persisters and infection: past, present, and progressing. Annu Rev Microbiol. 2019 Sep 8;73:359–385. doi: 10.1146/annurev-micro-020518-115650 31500532

[5] Fisher RA, Gollan B, Helaine S. Persistent bacterial infections and persister cells. Nat Rev Microbiol. 2017 Aug;15(8):453–464. doi: 10.1038/nrmicro.2017.42 28529326

[6] Levin-Reisman I, Ronin I, Gefen O, et al. Antibiotic tolerance facilitates the evolution of resistance. Science. 2017 Feb 24;355(6327):826–830. doi: 10.1126/science.aaj2191 28183996

[7] Cohen NR, Lobritz MA, Collins JJ. Microbial persistence and the road to drug resistance. Cell Host Microbe. 2013 Jun 12;13(6):632–642. doi: 10.1016/j.chom.2013.05.009 23768488 PMC3695397

[8] Liu J, Gefen O, Ronin I, et al. Effect of tolerance on the evolution of antibiotic resistance under drug combinations. Science. 2020 Jan 10;367(6474):200–204. 31919223 10.1126/science.aay3041

[9] Windels EM, Michiels JE, Fauvart M, et al. Bacterial persistence promotes the evolution of antibiotic resistance by increasing survival and mutation rates. ISME J. 2019 May;13(5):1239–1251. doi: 10.1038/s41396-019-0344-9 30647458 PMC6474225

[10] Fauvart M, De Groote VN, Michiels J. Role of persister cells in chronic infections: clinical relevance and perspectives on anti-persister therapies. J Med Microbiol. 2011 Jun;60(Pt 6):699–709. doi: 10.1099/jmm.0.030932-0 21459912

[11] Yan J, Bassler BL. Surviving as a community: antibiotic tolerance and persistence in bacterial biofilms. Cell Host Microbe. 2019 Jul 10;26(1):15–21. doi: 10.1016/j.chom.2019.06.002 31295420 PMC6629468

[12] Balaban NQ, Gerdes K, Lewis K, et al. A problem of persistence: still more questions than answers? Nat Rev Microbiol. 2013 Aug;11(8):587–591. doi: 10.1038/nrmicro3076 24020075

[13] Pu Y, Ke Y, Bai F. Active efflux in dormant bacterial cells – new insights into antibiotic persistence. Drug Resist Updat. 2017 Jan;30:7–14. doi: 10.1016/j.drup.2016.11.002 28363336

[14] Pu Y, Zhao Z, Li Y, et al. Enhanced efflux activity facilitates drug tolerance in dormant bacterial cells. Mol Cell. 2016 Apr 21;62(2):284–294. doi: 10.1016/j.molcel.2016.03.035 27105118 PMC4850422

[15] Dijkshoorn L, Nemec A, Seifert H. An increasing threat in hospitals: multidrug-resistant *Acinetobacter baumannii*. Nat Rev Microbiol. 2007 Dec;5(12):939–951. doi: 10.1038/nrmicro1789 18007677

[16] McConnell MJ, Actis L, Pachon J. *Acinetobacter baumannii*: human infections, factors contributing to pathogenesis and animal models. Fems Microbiol Rev. 2013 Mar;37(2):130–155. doi: 10.1111/j.1574-6976.2012.00344.x 22568581

[17] Antunes LC, Visca P, Towner KJ. *Acinetobacter baumannii*: evolution of a global pathogen. Pathog Dis. 2014;71(3):292–301. doi: 10.1111/2049-632X.12125 24376225

[18] Xie R, Zhang XD, Zhao Q, et al. Analysis of global prevalence of antibiotic resistance in *Acinetobacter baumannii* infections disclosed a faster increase in OECD countries. Emerg Microbes Infect. 2018 Mar 14;7(1):31. 29535298 10.1038/s41426-018-0038-9 PMC5849731

[19] WHO publishes list of bacteria for which new antibiotics are urgently needed 2017. Available from: http://www.who.int/mediacentre/news/releases/2017/bacteria-antibiotics-needed/en/

[20] Barth VC Jr., Rodrigues BA, Bonatto GD, et al. Heterogeneous persister cells formation in *Acinetobacter baumannii*. PLoS One. 2013;8(12):e84361. doi: 10.1371/journal.pone.0084361 24391945 PMC3877289

[21] Alkasir R, Ma Y, Liu F, et al. Characterization and transcriptome analysis of *Acinetobacter baumannii* persister cells. Microb Drug Resist. 2018 Dec;24(10):1466–1474. doi: 10.1089/mdr.2017.0341 29902105

[22] Nicol M, Mlouka MAB, Berthe T, et al. Anti-persister activity of squalamine against *Acinetobacter baumannii*. Int J Antimicrob Agents. 2019 Mar;53(3):337–342. doi: 10.1016/j.ijantimicag.2018.11.004 30423343

[23] Kaur A, Sharma P, Capalash N. Curcumin alleviates persistence of *Acinetobacter baumannii* against colistin. Sci Rep. 2018 Jul 23;8(1):11029. doi: 10.1038/s41598-018-29291-z 30038318 PMC6056455

[24] Donamore BK, Gallo SW, Abreu Ferreira PM, et al. Levels of persisters influenced by aeration in *Acinetobacter calcoaceticus-baumannii*. Future Microbiol. 2018 Feb;13:209–219. doi: 10.2217/fmb-2017-0153 29302999

[25] Zheng J, Tung SL, Leung KY. Regulation of a type III and a putative secretion system in Edwardsiella tarda by EsrC is under the control of a two-component system, EsrA-EsrB. Infect Immun. 2005 Jul;73(7):4127–4137. doi: 10.1128/IAI.73.7.4127-4137.2005 15972502 PMC1168592

[26] Basler M, Pilhofer M, Henderson GP, et al. Type VI secretion requires a dynamic contractile phage tail-like structure. Nature. 2012 Feb 26;483(7388):182–186. doi: 10.1038/nature10846 22367545 PMC3527127

[27] Zou J, Zhang W, Zhang H, et al. Studies on aminoglycoside susceptibility identify a novel function of KsgA to secure translational fidelity during antibiotic stress. Antimicrob Agents Chemother. 2018 Oct;62(10):e853–e818. doi: 10.1128/AAC.00853-18 PMC6153849 30082289

[28] Peleg AY, Jara S, Monga D, et al. *Galleria mellonella* as a model system to study *Acinetobacter baumannii* pathogenesis and therapeutics. Antimicrob Agents Chemother. 2009 Jun;53(6):2605–2609. doi: 10.1128/AAC.01533-08 19332683 PMC2687231

[29] Kawai Y, Mickiewicz K, Errington J. Lysozyme counteracts beta-lactam antibiotics by promoting the emergence of L-form bacteria. Cell. 2018 Feb 22;172(5):1038–1049. e10. doi: 10.1016/j.cell.2018.01.021 29456081 PMC5847170

[30] Khalil MAF, Moawad SS, Hefzy EM. In vivo activity of co-trimoxazole combined with colistin against *Acinetobacter baumannii* producing OXA-23 in a *Galleria mellonella* model. J Med Microbiol. 2019 Jan;68(1):52–59. doi: 10.1099/jmm.0.000872 30422109

[31] Brauner A, Fridman O, Gefen O, et al. Distinguishing between resistance, tolerance and persistence to antibiotic treatment. Nat Rev Microbiol. 2016 Apr;14(5):320–330. doi: 10.1038/nrmicro.2016.34 27080241

[32] Harms A, Maisonneuve E, Gerdes K. Mechanisms of bacterial persistence during stress and antibiotic exposure. Science. 2016 Dec 16;354(6318):aaf4268. doi: 10.1126/science.aaf4268 27980159

[33] Dorr T, Davis BM, Waldor MK. Endopeptidase-mediated beta lactam tolerance. PLoS Pathog. 2015 Apr;11(4):e1004850. doi: 10.1371/journal.ppat.1004850 25884840 PMC4401780

[34] Cardoso K, Gandra RF, Wisniewski ES, et al. Dnak and GroEL are induced in response to antibiotic and heat shock in *Acinetobacter baumannii*. J Med Microbiol. 2010 Sep;59(Pt 9):1061–1068. doi: 10.1099/jmm.0.020339-0 20576751

[35] Kuru E, Tekkam S, Hall E, et al. Synthesis of fluorescent D-amino acids and their use for probing peptidoglycan synthesis and bacterial growth in situ. Nat Protoc. 2015 Jan;10(1):33–52. doi: 10.1038/nprot.2014.197 25474031 PMC4300143

[36] Hornsey M, Wareham DW. In vivo efficacy of glycopeptide-colistin combination therapies in a *Galleria mellonella* model of *Acinetobacter baumannii* infection. Antimicrob Agents Chemother. 2011 Jul;55(7):3534–3537. doi: 10.1128/AAC.00230-11 21502628 PMC3122470

[37] Hornsey M, Phee L, Longshaw C, et al. In vivo efficacy of telavancin/colistin combination therapy in a *Galleria mellonella* model of *Acinetobacter baumannii* infection. Int J Antimicrob Agents. 2013 Mar;41(3):285–287. doi: 10.1016/j.ijantimicag.2012.11.013 23312607

[38] Gupta S, Govil D, Kakar PN, et al. Colistin and polymyxin B: a re-emergence. Indian J Crit Care Med. 2009 Apr–Jun;13(2):49–53. doi: 10.4103/0972-5229.56048 19881183 PMC2772240

[39] Loho T, Dharmayanti A. Colistin: an antibiotic and its role in multiresistant gram-negative infections. Acta Med Indones. 2015 Apr;47(2):157–168. 26260559

[40] Falla TJ, Chopra I. Joint tolerance to beta-lactam and fluoroquinolone antibiotics in *Escherichia coli* results from overexpression of hipA. Antimicrob Agents Chemother. 1998 Dec;42(12):3282–3284. doi: 10.1128/AAC.42.12.3282 9835528 PMC106036

[41] Hall AM, Gollan B, Helaine S. Toxin-antitoxin systems: reversible toxicity. Curr Opin Microbiol. 2017 Apr;36:102–110. doi: 10.1016/j.mib.2017.02.003 28279904

[42] Hansen S, Vulic M, Min J, et al. Regulation of the *Escherichia coli* HipBA toxin-antitoxin system by proteolysis. PLoS One. 2012;7(6):e39185. doi: 10.1371/journal.pone.0039185 22720069 PMC3376134

[43] Tsilibaris V, Maenhaut-Michel G, Van Melderen L. Biological roles of the Lon ATP-dependent protease. Res Microbiol. 2006 Oct;157(8):701–713. doi: 10.1016/j.resmic.2006.05.004 16854568

[44] Germain E, Castro-Roa D, Zenkin N, et al. Molecular mechanism of bacterial persistence by HipA. Mol Cell. 2013 Oct 24;52(2):248–254. doi: 10.1016/j.molcel.2013.08.045 24095282

[45] Semanjski M, Germain E, Bratl K, et al. The kinases HipA and HipA7 phosphorylate different substrate pools in *Escherichia coli* to promote multidrug tolerance. Sci Signal. 2018 Sep 11;11(547):eaat5750. doi: 10.1126/scisignal.aat5750 30206139

[46] Ronneau S, Helaine S. Clarifying the link between toxin-antitoxin modules and bacterial persistence. J Mol Biol. 2019 Aug 23;431(18):3462–3471. doi: 10.1016/j.jmb.2019.03.019 30914294

[47] Pu Y, Li Y, Jin X, et al. ATP-dependent dynamic protein aggregation regulates bacterial dormancy depth critical for antibiotic tolerance. Mol Cell. 2019 Jan 3;73(1):143–156. e4. doi: 10.1016/j.molcel.2018.10.022 30472191

[48] Shan Y, Brown Gandt A, Rowe SE, et al. ATP-dependent persister formation in *Escherichia coli*. MBio. 2017 Feb 7;8(1):e02267–16. doi: 10.1128/mBio.02267-16 28174313 PMC5296605

[49] Errington J, Mickiewicz K, Kawai Y, et al. L-form bacteria, chronic diseases and the origins of life. Philos Trans R Soc Lond B Biol Sci. 2016 Nov 5;371(1707):20150494. doi: 10.1098/rstb.2015.0494 27672147 PMC5052740

[50] Monahan LG, Turnbull L, Osvath SR, et al. Rapid conversion of *Pseudomonas aeruginosa* to a spherical cell morphotype facilitates tolerance to carbapenems and penicillins but increases susceptibility to antimicrobial peptides. Antimicrob Agents Chemother. 2014;58(4):1956–1962. doi: 10.1128/AAC.01901-13 24419348 PMC4023726

[51] Roberts D, Higgs E, Rutman A, et al. Isolation of spheroplastic forms of *Haemophilus influenzae* from sputum in conventionally treated chronic bronchial sepsis using selective medium supplemented with N-acetyl-D-glucosamine: possible reservoir for re-emergence of infection. Br Med J (Clin Res Ed). 1984 Nov 24;289(6456):1409–1412. doi: 10.1136/bmj.289.6456.1409 PMC1443692 6437576

[52] Cross T, Ransegnola B, Shin JH, et al. Spheroplast-mediated carbapenem tolerance in gram-negative pathogens. Antimicrob Agents Chemother. 2019 Sep;63(9):e00756–19. doi: 10.1128/AAC.00756-19 31285232 PMC6709500

[53] Rojas ER, Billings G, Odermatt PD, et al. The outer membrane is an essential load-bearing element in gram-negative bacteria. Nature. 2018 Jul;559(7715):617–621. doi: 10.1038/s41586-018-0344-3 30022160 PMC6089221

[54] Osawa M, Erickson HP. L form bacteria growth in low-osmolality medium. Microbiology. 2019 Aug;165(8):842–851. doi: 10.1099/mic.0.000799 30958258 PMC7008213

[55] Mercier R, Kawai Y, Errington J. Excess membrane synthesis drives a primitive mode of cell proliferation. Cell. 2013 Feb 28;152(5):997–1007. doi: 10.1016/j.cell.2013.01.043 23452849

[56] Chung ES, Ko KS. Eradication of persister cells of *Acinetobacter baumannii* through combination of colistin and amikacin antibiotics. J Antimicrob Chemother. 2019 May 1;74(5):1277–1283. doi: 10.1093/jac/dkz034 30759206

[57] Ji X, Zou J, Peng H, et al. Alarmone Ap4A is elevated by aminoglycoside antibiotics and enhances their bactericidal activity. Proc Natl Acad Sci U S A. 2019 May 7;116(19):9578–9585. doi: 10.1073/pnas.1822026116 31004054 PMC6511005

