# Supplementary material for: Non-walled spherical Acinetobacter baumannii is an important type of persister upon β-lactam antibiotic treatment
Source: Emerg Microbes Infect. 2020 Jun 2;9(1):1149–59. doi: 10.1080/22221751.2020.1770630 (PMC7448848; doi:10.1080/22221751.2020.1770630)
Supplement: Supplemental Material [file TEMI_A_1770630_SM3004.zip › Zou et al. supl_20200503_EMI.docx]

**Non-walled spherical *Acinetobacter baumannii* is an important type of persisters upon β-lactam antibiotics treatment**

Jin Zou^1^, Si-Hoi Kou^1^, Ruiqiang Xie^1^, Michael S. VanNieuwenhze^2^, Jiuxin Qu^3^, Bo Peng^4^ and Jun Zheng^1,5 *^

**Affiliations:**

^1^ Faculty of Health Sciences, University of Macau, Macau SAR, China;

^2^ Department of Molecular and Cellular Biochemistry, Indiana University, Bloomington, IN  47405-7003, USA;

^3^ Department of Clinical Laboratory, The Third People's Hospital of Shenzhen, Southern University of Science and Technology, National Clinical Research Center for Infectious Diseases, Shenzhen, China;

^4^ School of Life Sciences, Sun Yat-sen University, Guangzhou 510006, China; Laboratory for Marine Biology and Biotechnology, Qingdao National Laboratory for Marine Science and Technology, Qingdao 266071, China;

^5^ Institute of Translational Medicine, University of Macau, Macau SAR, China.

^*^ Correspondence should be addressed to:

Jun Zheng, [junzheng@um.edu.mo](mailto:junzheng@um.edu.mo)

**Running title:** Non-walled *Acinetobacter baumannii* is persister

**Keywords:** *Acinetobacter baumannii*, persisters, drug tolerance, non-walled spherical bacterium, β-lactam antibiotics

**Supporting experimental procedures**

**Isolation of bacterial persisters and the killing assay.** Frozen stocks of *A. baumannii* ATCC17978 were inoculated into LB medium and grew at 37°C. Overnight bacteria were sub-cultured at 1:20 into 4 ml of fresh medium so that the initial cell density was about 2 ~ 4 × 10^8^ cells/ml. Antibiotics were directly added to the diluted culture to a ﬁnal concentration as indicated in the text. Treated bacteria were collected at different time-points post treatment (0, 2, 4, 6 and 24h) and washed twice with ﬁltered 1× phosphate-buffered saline (PBS). Bacteria were then serially diluted by 1× PBS and spotted on an LB agar plate for enumeration. To test combinational killing efficiency of antibiotics, bacteria were inoculated into cation-adjusted Mueller-Hinton broth (MHB) medium and cultured at 37°C for about 12 h. Overnight culture was diluted at 1:100 into fresh media and grown at 37°C to OD_600_ ≈ 0.3 - 0.4. Antibiotics were then added to a ﬁnal concentration as indicated in the text. The survival cells were enumerated as described above.

**Cell staining.** To identify live cells in the bacterial population, we used SYTO-9 and PI from LIVE/DEAD BacLight Bacterial Viability Kits (Invitrogen) to stain bacteria following the product user manual. Briefly, persister cells were obtained as described above and collected at 8,000 rpm for 3 min. The cell pellets were resuspended in 1 mL of 0.85% NaCl and added with SYTO-9 and PI at the final concentration of 5 μM and 30 μM, respectively. The suspension was then mixed thoroughly and incubated at room temperature for 15 min, protected from light. Ten microliter of bacteria were then immobilized by poly-L-lysine onto coverslips for fluorescence imaging. The HADA staining followed the procedures as described previously [1]. Briefly, the overnight bacterial culture was diluted at 1:1000 into fresh LB medium supplemented with 50 μM HADA and incubated in the dark at 37℃ for bacterial growth. Cells incorporating HADA were diluted at 1:20 into fresh medium in the presence of 50 μM of HADA. Antibiotics were added for killing. At 4 h post killing, the survivors were collected and washed by 1 × PBS for three times. The cells were used for fluorescent microscopy as describe above.

**Time-lapse microscopy of antibiotic killing and persister regrowth.** For the killing process, bacteria cultured overnight was collected, washed once with 1 × PBS and then diluted at 1:10 in 1 × PBS. Cell suspension was then added into chamber of FCS2 and incubated at room temperature for 15 min. The chamber was coated with poly-L-lysine for 10 min at room temperature and then rinsed once with 1 × PBS before the addition of cell suspension. After rinsed with 1 × PBS, 300 μL of fresh LB medium supplemented with 1% methylcellulose and 10 μg/ml meropenem were added into the chamber. The flow system was then assembled for microscopy at 37℃.

To record the regrowth of persisters, the bacteria grown overnight were subcultured at 1:20 and meropenem was added to a final concentration of 10 μg/ml. After growth at 37℃ for 4 h in shaking incubator, 3 ml of the culture was pelleted at 5,000 rpm, washed once and then re-suspended in 300 μL of 1 × PBS supplemented with 1 mM CaCl_2_. The isolated persisters were then added into poly-L-lysine coated chamber of FCS2 and incubated at room temperature for 10 min. After rinsing with 1 × PBS once, fresh LB medium supplemented with 2% methylcellulose was added into the chamber, and the equipment was then assembly for microscopy.

***G. mellonella* infection model.** Briefly, fifteen randomly chosen caterpillars which weight ranging from 300 to 400 mg were employed in each group of the assays. Overnight culture of *dnak*::*msfGFP* was collected and re-suspended in 1 × PBS. Bacteria were diluted at 1:10 and washed once with 1 × PBS before the infection to larvae. An aliquot of 10 μL of the bacteria (~5 x 10^6^ CFU) was inoculated into the hemocoel of each caterpillar via the last left proleg. After injection, caterpillars were placed in the clean dish and incubated at 37℃. At the certain time-point post infection as indicated in the text, the live larvae that did not show melanisation and displayed movement in response to touch were put in 1.5mL Eppendorf tube and incubated on ice for 30 min. Two posterior segments were then cut off with surgical scissors and the haemolymph was extracted into the clean microfuge tubes. One microliter of the hemolymph was added onto a 3% agarose pad for microscopy. For antibiotic treatment experiment, the larvae were infected with bacteria and incubated at 37℃ for 1 h, and meropenem were then administered by injection at dose of 6 mg/kg into a different proleg within 30 min. The larvae were kept at 37℃ for another 30 min. The live worms were sacrificed and the haemolymph were observed under a microscope as described above.

**Minimum inhibitory concentration degermation (MIC_50_).** The MIC_50_ of each antibiotic was performed following previous description using the 2-fold serial microtiter broth dilution method [2]. Overnight bacterial culture was diluted to OD_600_ about 0.02, and 100 µl diluted bacteria were added into each well containing serial diluted antibiotics. The plate was incubated without shaking at 37℃ for 24 h and then OD_600_ was measured, and the MIC_50_ values were calculated using Graphpad Prism 6.

**Determination of the fractional inhibitory concentration index (FICI).** FICI determination was performed following previous description [3]. Briefly, seven serial, two-fold dilution, of each antibiotic were prepared in 1.5 mL of Eppendorf tubes to obtain four-time of the final concentrations, which ranged from 3.125 to 200 μg/mL for ampicillin, 1.563 to 100 μg/mL for carbenicillin, 0.02 to 1.25 μg/mL for meropenem and 0.195 to 12.5 μg/mL for colistin. In a 96-well plate, the first horizontal and vertical rows were left with only one agent, and 50 μL of each dilution of colistin was added in the following each vertical row, and 50 μL of other antibiotic dilution was added in the following each horizontal row to obtain three different 8-by-8 checkerboard designs: colistin plus ampicillin, colistin plus carbenicillin, and colistin plus meropenem. After inoculation with 100 μL of bacteria with OD_600_ about 0.02 (about 1 × 10^6^ CFU/mL bacteria), the plates were incubated at 37℃ for 24 h and measured optical density at 600 nm. FICI (ΣFIC) for the combination of antibitoics was calculated using the formula ΣFIC = FIC_A_ + FIC_B_ = (C_A_/MIC_A_) + (C_B_/MIC_B_), where MIC_A_ and MIC_B_ are the MICs of drugs A and B alone, respectively, and C_A_ and C_B_ are the concentrations of the drugs in combination, respectively. Percent growth of each well was calculated based on the optical density (OD) as (ODwell − ODbackground)/ (ODdrug-free well − ODbackground) ×100%. MICs of drug alone and combination were defined as the lowest concentrations showing <10% of the growth of no drug control. The results were interpreted as follows: FICI ≤ 0.5 synergistic effect and FICI > 0.5 no synergistic effect [4].

**Supporting figures and legends**


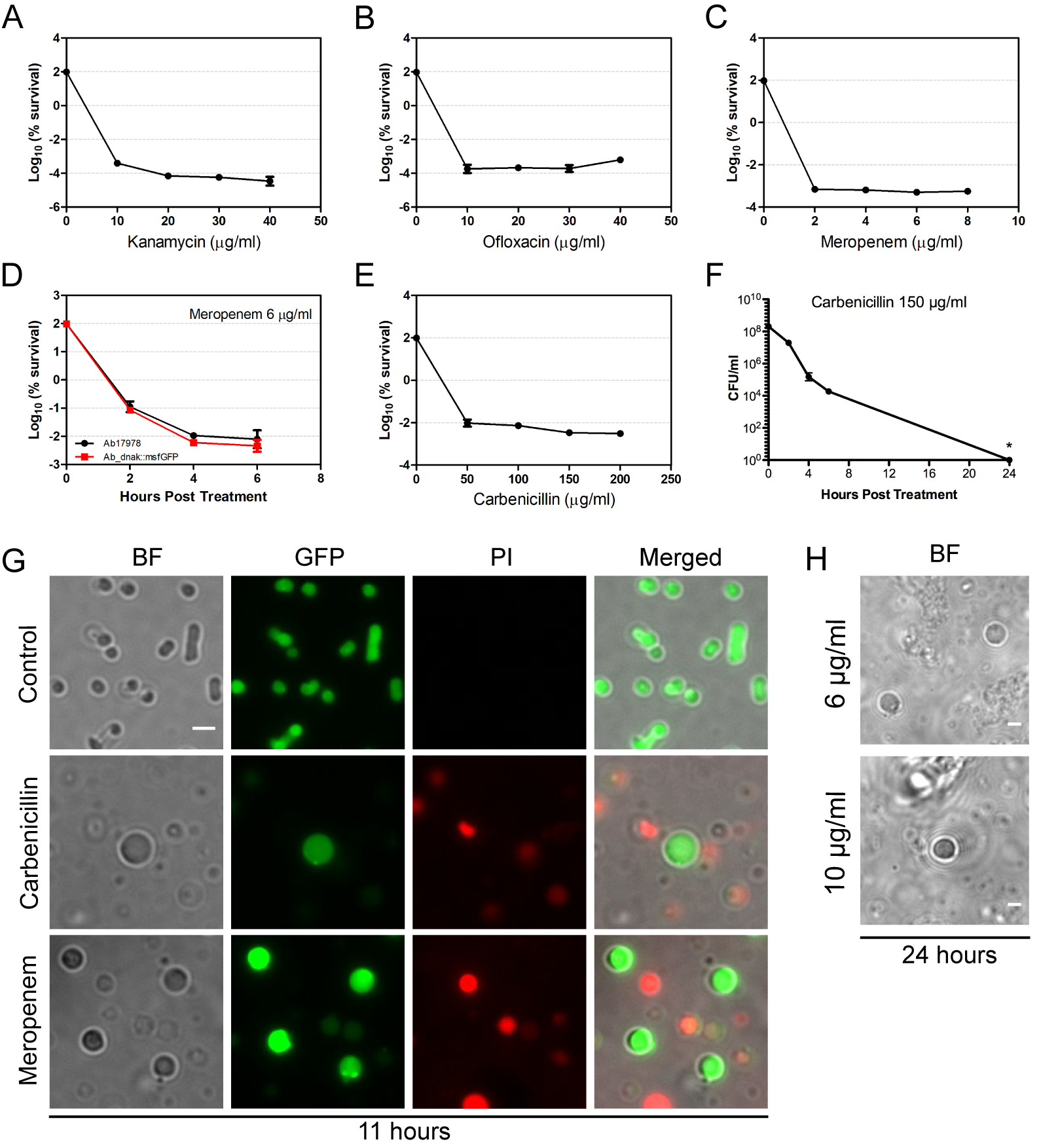


**Fig. S1.** (A-C) The survival of *A. baumannii* ATCC17978 treated by increasing concentrations of meropenem (A), kanamycin (B) and ofloxacin (C). (D) The survival of *A. baumannii* ATCC17978 and *dnaK::msfGFP* under the treatment of 6 μg/ml of meropenem. (E) The survival of *A. baumannii* ATCC17978 treated by increasing concentrations of carbenicillin. (F) The survival of *A. baumannii* ATCC17978 under the treatment of 150 μg/ml of carbenicillin. *: under detect limitation. (G) Fluorescent imaging of the survivors of *dnaK::msfGFP* from the treatment by 150 μg/ml of carbenicillin or 6 μg/ml of meropenem for 11 hr. (H) The survivors of *A. baumannii* ATCC17978 from the treatment by 6 μg/ml (upper) or 10 μg/ml of meropenem (lower) for 24 hr.


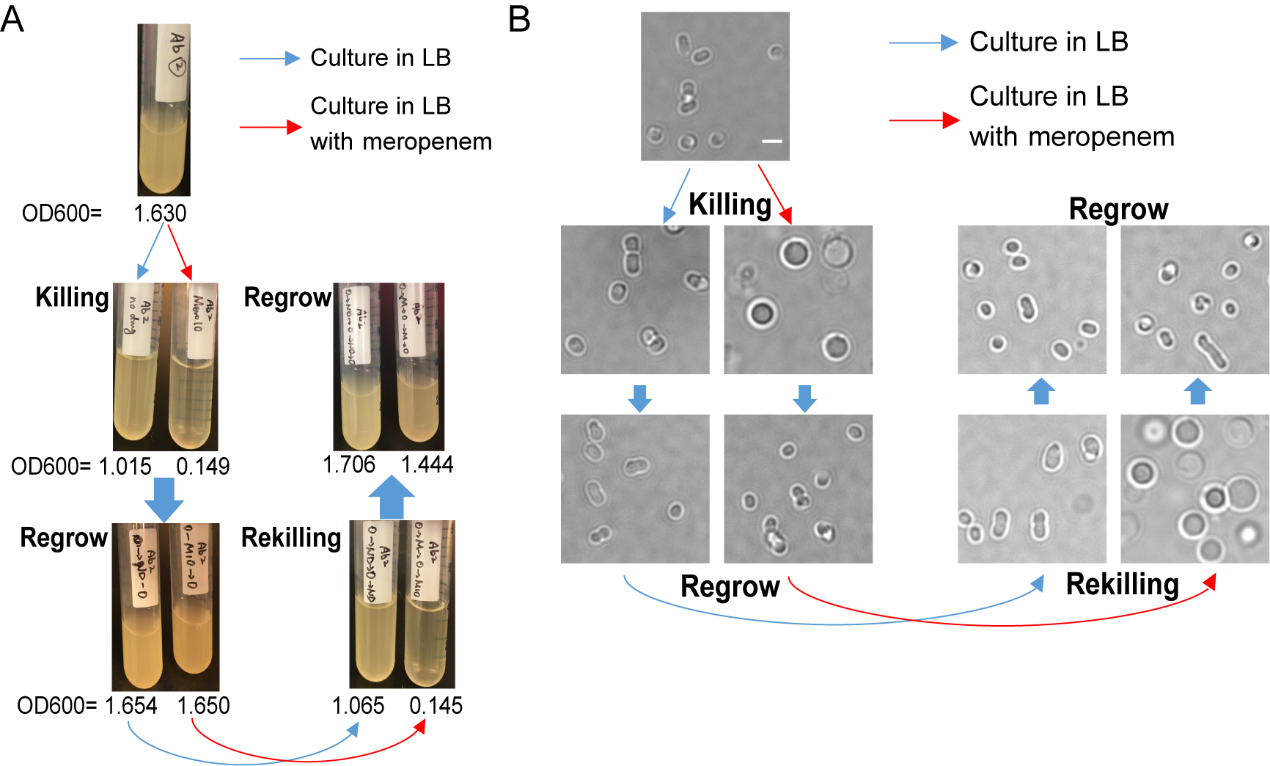


**Fig. S2.** *In vitro* drug tolerance and regrowth assay showing that *A. baumannii* is persistence rather than resistance against meropenem. (A) The overnight bacteria were subcultured at 1:20 into fresh LB without or with 10 μg/ml of meropenem. Both cultures were grown at 37℃ for 4 h to get persistent cells. These cells were collected by centrifugation and re-suspended in fresh LB to resume growth. The killing and regrowth process were repeated. (B) The morphotype of bacteria from killing and regrowth process of (A).


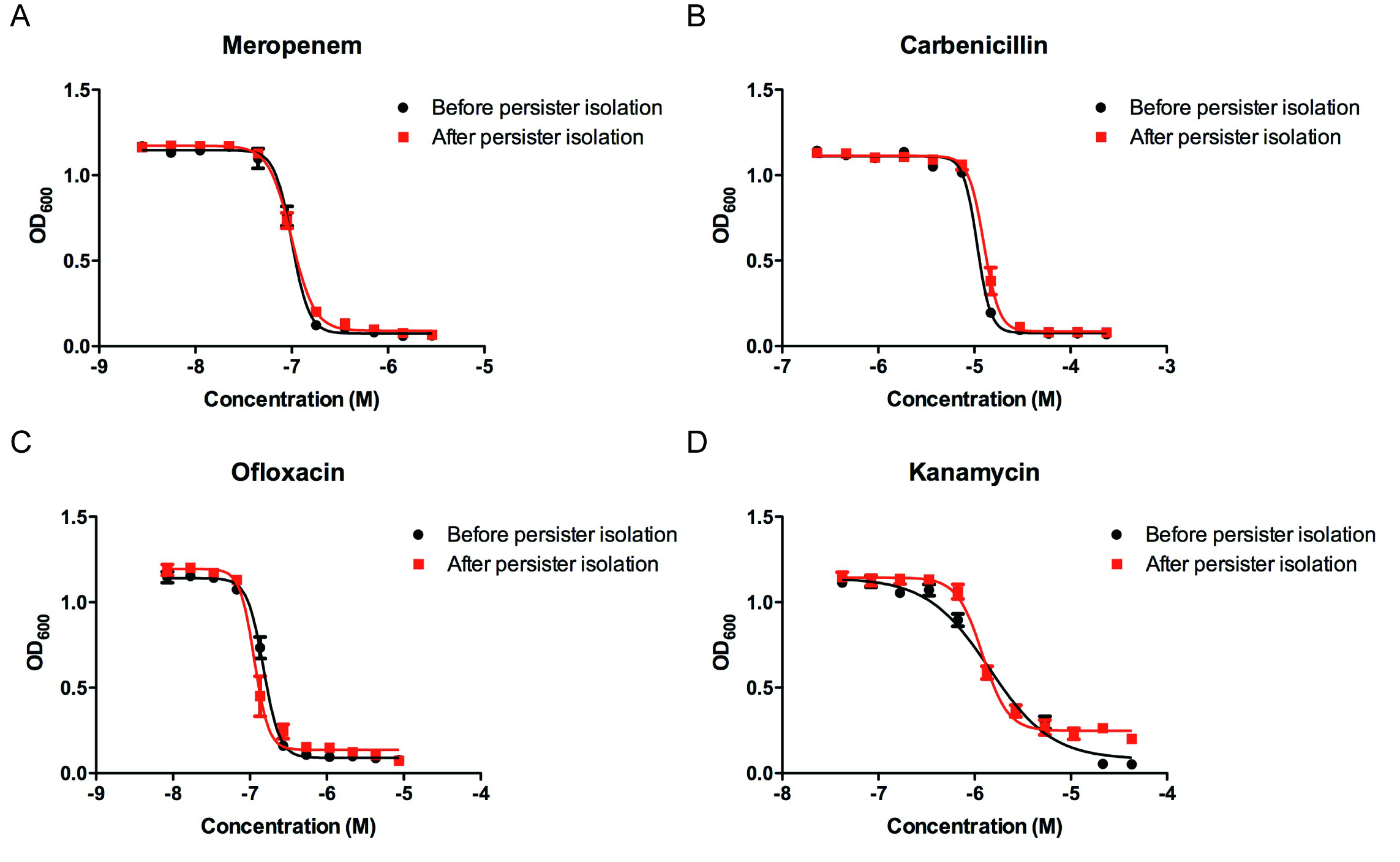


**Fig. S3.** The minimum inhibitory concentration test for each antibiotic before and after persisters isolation. The value of MIC_50_ of each antibiotic was shown in Table S3.


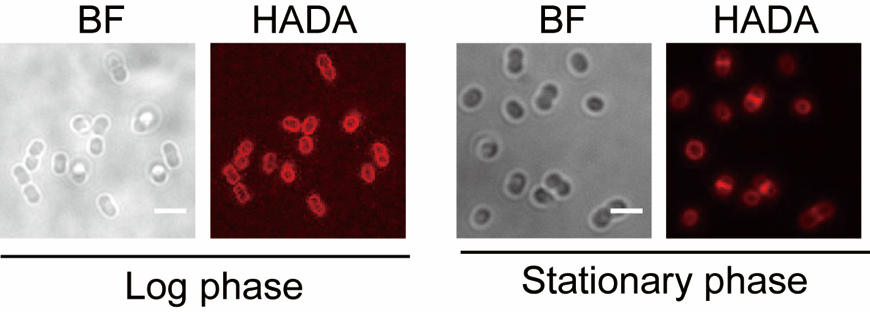


**Fig. S4.** Fluorescent microscopy of *A. baumannii* ATCC17978 in logarithmic phase and stationary phase stained with HADA.


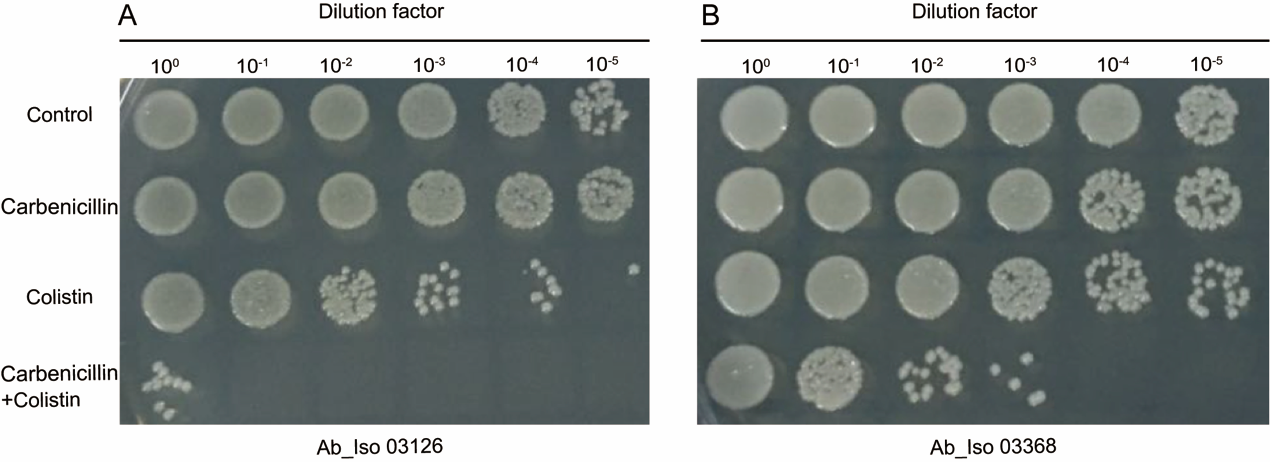


**Fig. S5.** Two clinical isolates of *A. baumannii* Iso 03126 (A) and Iso 03368 (B) were treated with 30 μg/ml carbenicillin and 2 μg/ml colistin alone, and their combination for 4 h.


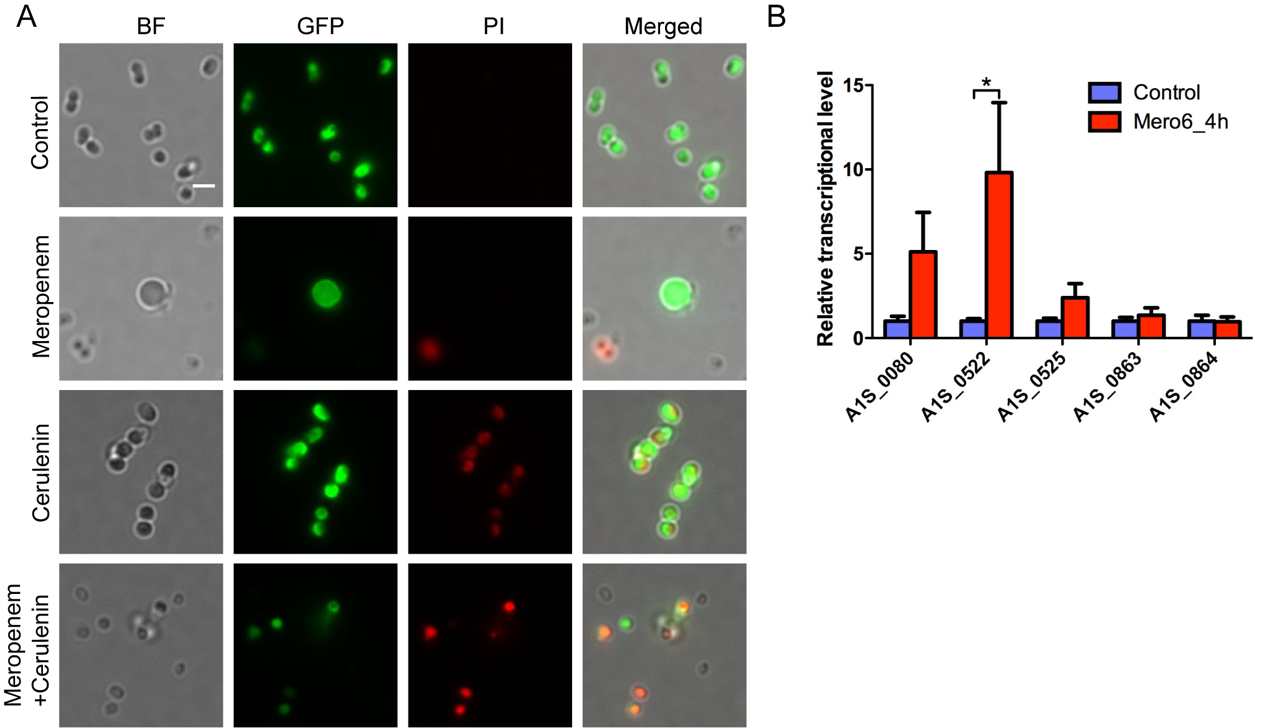


**Fig. S6 Inhibition on fatty acid biosynthesis prevents the formation of spherical *A. baumannii*.** (A) The fluorescent microscopy showing that cerulenin treatment prevented the formation of spherical cells. *dnaK::msfGFP* were treated without or with 10 μg/ml cerulenin, or 6 μg/ml meropenem, or the combination of cerulenin and meropenem at 37℃ for 4 h, followed by PI staining for microscopy. (Scale bar, 2μm). (B) Relative transcriptional expression of genes encoding 3-ketoacyl-ACP synthases in both bacterial cells with or without antibiotic treatment. The values represent the mean ± SD from three independent experiments. (*: p value <0.05).

**Table S1.** Strains and plasmids used in this study.

| **Strain / plasmid** | **Genotype or description** | **Source or reference** |
| --- | --- | --- |
| **Strains** |  |  |
| ATCC17978 | *A. baumannii* wild type | ATCC |
| *dnak*::*msfGFP* | *A. baumannii* ATCC17978  *msfGFP* fusion at the C-terminal of *dnaK* in chromosome | This study |
| MG1655+pACYC184-eGFP | MG1655 with pACYC184-eGFP | [5] |
| **Plasmids** |  |  |
| pEXG2 | ColE1 mob+, *sacB* suicide vector (Gm^R^) | [6] |
| pEXG2-*dnak*::*gfp* | pEXG2 with a *A. baumannii* wild type *dnaK* in frame with *msf*GFP gene at the C-terminal | This study |

**Table S2.** Primers used in this study.

| **Primers** | **Sequence (5’-3’)** | **Description** |
| --- | --- | --- |
| 16S_338F | ACTCCTACGGGAGGCAGCAG | For qRT-PCR of 16S rDNA |
| 16S_518R | ATTACCGCGGCTGCTGG |  |
| qRT_A1S_0080_FOR | GGGTACGCTGCCAATAGTGA | For qRT-PCR of A1S_0080 |
| qRT_A1S_0080_REV | GTTGAAGTGCCATGCGTGTT |  |
| qRT_A1S_0522_FOR | GTGGCGTCGATACCTTGTGT | For qRT-PCR of A1S_0522 |
| qRT_A1S_0522_REV | CCGCCCCCATTAACATGACA |  |
| qRT_A1S_0525_FOR | GGTGCGGCAGTTTTTGATGT | For qRT-PCR of A1S_0525 |
| qRT_A1S_0525_REV | CCTCGTGCTTTGGCATGTTC |  |
| qRT_A1S_0863_FOR | TGTCAGCACCGTCACTGTTT | For qRT-PCR of A1S_0863 |
| qRT_A1S_0863_REV | CCGCGACGGTTTCGTTATTG |  |
| qRT_A1S_0864_FOR | TGCACATACGCCCATGAACC | For qRT-PCR of A1S_0864 |
| qRT_A1S_0864_REV | CGGGACACGTTTTAACCCAA |  |
| DnaK_L_REV | CGGCCGCttttttgtcatcttttacttcagtgaactcagcatc | For construction of *dnaK::msfGFP* |
| pEXG2_DnaK_L_FOR | ccggggatcctctagcttgtacaacaaaaagtacaagaattcttcggtag |  |
| DnaK_R_FOR | gatgagctctacaaataatttgtttctttaaaaaagaccgcgc |  |
| pEXG2_DnaK_R_REV | ctgcaggtcgactctaggctatgttgagaaaactttgtttgtaattctaacga |  |
| homL_GFP_FOR | acaaaaaaGCGGCCGCAGGAGGAGGATC |  |
| homR_GFP_REV | aacaaattatttgtagagctcatccatgccgTGCGT |  |
| Iden_Lout_FOR | cgtaactcgtgcaaaacttgaaggt | For conformation of *dnaK::msfGFP* |
| Iden_Rout_REV | ttgagccagaagtcgtgctca |  |
| Iden_GFPin_FOR | CTCTGTTCGTGGTGAAGGTGAAGG |  |
| Iden_GFPin_REV | GGCAGCAGAACCGGACCATC |  |

**Table S3.** MIC_50_ for each antibiotic before and after persister isolation.

**
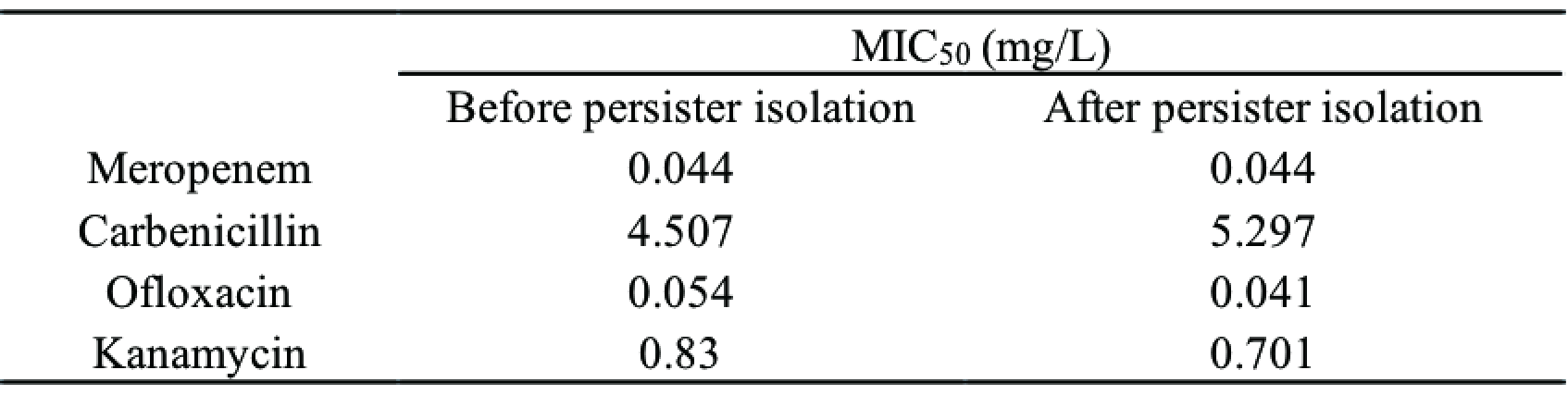
**

**Table S4.** FIC index of drugs combination against *A. baumannii* ATCC17978.


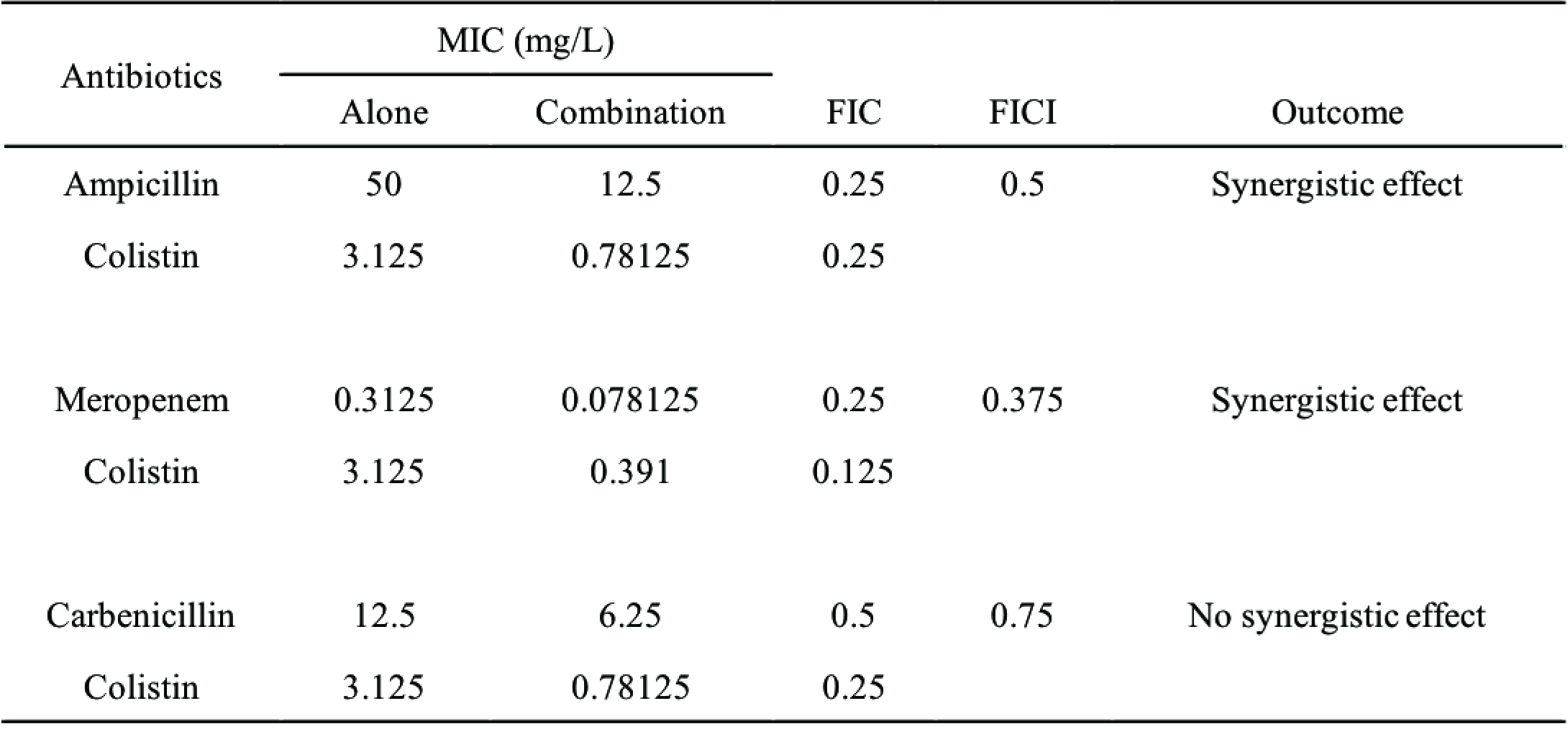


**References**

1. Kuru E, Tekkam S, Hall E, et al. Synthesis of fluorescent D-amino acids and their use for probing peptidoglycan synthesis and bacterial growth in situ. Nat Protoc. 2015 Jan;10(1):33-52.

2. Jorgensen JH, Turnidge JD. Susceptibility test methods: dilution and disk diffusion methods. Manual of Clinical Microbiology, Eleventh Edition: American Society of Microbiology; 2015. p. 1253-1273.

3. Meletiadis J, Pournaras S, Roilides E, et al. Defining fractional inhibitory concentration index cutoffs for additive interactions based on self-drug additive combinations, Monte Carlo simulation analysis, and in vitro-in vivo correlation data for antifungal drug combinations against Aspergillus fumigatus. Antimicrob Agents Chemother. 2010 Feb;54(2):602-9.

4. Odds FC. Synergy, antagonism, and what the chequerboard puts between them. J Antimicrob Chemother. 2003 Jul;52(1):1.

5. Zou J, Zhang W, Zhang H, et al. Studies on Aminoglycoside Susceptibility Identify a Novel Function of KsgA To Secure Translational Fidelity during Antibiotic Stress. Antimicrob Agents Chemother. 2018 Oct;62(10).

6. Rietsch A, Vallet-Gely I, Dove SL, et al. ExsE, a secreted regulator of type III secretion genes in Pseudomonas aeruginosa. Proc Natl Acad Sci U S A. 2005 May 31;102(22):8006-11.
